# The Intention of Sports Participants to Utilize Digital Technology for Engagement: The Moderating Role of Self-Efficacy

**DOI:** 10.3390/bs15030367

**Published:** 2025-03-14

**Authors:** Rubin Qian, Kitak Kim

**Affiliations:** Department of Leisure Service Sports, Pai Chai University, Daejeon 35345, Republic of Korea; ghggg9786@gmail.com

**Keywords:** digital technology, sports participation, behavioral intention, BRT, self-efficacy

## Abstract

Digital technology has greatly influenced the way the public engages in sports activities. However, the behavioral decision-making process associated with the adoption of digital technology for sports participation remains unclear. This study employs the Behavioral Reasoning Theory to elucidate the cognitive processes underlying participants’ behavioral intentions by examining the reasons for and against the adoption, as well as the influence of self-efficacy. The model presented is a theoretical expansion of the current innovation frameworks within sports management and marketing. The findings indicate that the reasons against adoption by respondents exert a more substantial impact on their attitudes and behavioral intentions than the reasons for adoption. Perceived enjoyment and perceived barriers to use are identified as the leading factors for reasons in favor of and against adoption, respectively. The study also highlights the pivotal role of participants’ self-efficacy in the cognitive decision-making process concerning the adoption of digital technology for sports participation. To amplify the impact of digital technology within the sports domain, it delineates the reasons for and against adoption and to account for the influence of self-efficacy in the decision-making process, thereby ensuring that the integration of digital technology with sports more effectively addresses the practical needs of participants.

## 1. Introduction

In the post epidemic era, new factors of production based on advanced digital technologies will play an important role in solving economic growth challenges (C. Jin et al., 2023). As an umbrella term, digital technologies encompass emerging modern innovations such as artificial intelligence (AI), big data, and the Internet of Things (IoT) (Qian & Kim, 2024). Sports, as a special social activity, expands the application scenarios of digital technologies. For example, artificial intelligence (AI) in sports can be utilized to develop personalized training systems, conduct comprehensive performance assessments, and deliver intelligent coaching frameworks (Keiper et al., 2023). Virtual reality (VR)-enhanced sports platforms, such as screen-based golf simulations, reduce participation costs and mitigate disruptions caused by external constraints (e.g., screen golf) (Seong & Hong, 2022). Similarly, intelligent interactive platforms enable real-time monitoring of athletic performance and enhance fan engagement opportunities (Uhrich, 2022), collectively fostering increased user participation in sports activities. Sports participation manifests in two dimensions: ameliorating structural and functional impairments or activity limitations through physical engagement, and serving a pivotal role in enhancing and sustaining physical and mental well-being. At the same time, the application of digital technologies allows for detailed records of participants’ training progress (Lupton, 2020; Tjønndal, 2022), thereby improving participants’ overall health and athleticism (J. P. Higgins, 2016). To some extent, it has been shown that digitization has greatly enriched the way in which participation in sport is carried out (Westmattelmann et al., 2021). Human behavior is intricate and diverse (Box-Steffensmeier et al., 2022), e.g., Bandura (1977) first introduced the concept of self-efficacy in 1977 and identified that self-efficacy influences individual behavior in four areas: behavioral choices, motivational effort, cognitive processes, and affective processes (W. Zhou & Guo, 2006) which implies that human behavior is influenced at multiple levels. The impact of technology on sports will lead to significant changes in future sports consumption (Frevel et al., 2022), a gap persists in the behavioral decision-making process of sports participants employing digital technology for sports engagement. Nevertheless, understanding whether and why target audiences embrace innovations remains critical for companies developing and marketing new products and services (Claudy et al., 2015).

To precisely comprehend the decision-making processes of consumers across various contexts, Westaby (2005) developed and validated the Behavioral Reasoning Theory (hereinafter referred to as BRT). The theory posits that individuals evaluate behavioral choices by considering whether new products or technologies enhance or hinder their actions, primarily accounting for the reasons supporting or opposing the adoption of new innovations. It is essential to consider both sets of reasons when analyzing the decision-making process (Dhir et al., 2021), as they can trigger different behaviors (Gupta & Arora, 2017b). Therefore, various fields have utilized this theory to analyze specific decision-making processes (Peterson & Simkins, 2019; Pillai & Sivathanu, 2018; Sivathanu, 2018a, 2018b). In the realm of sports, Uhrich (2022) found that previous research within the context of sports consumption (Ha et al., 2015; Kim et al., 2017) only revealed factors supporting the adoption of new technology, but neglected the factors resisting innovation. Although the development of digital technology is actively influencing the lifestyles and behaviors of people worldwide (Luo & He, 2021), it is still unclear what sports participants think about using digital technology for sports participation and how it affects their decision-making, because comprehensive benchmark data on technology adoption trends and perceived impacts across various domains within the field of sports remains limited (Qi et al., 2024).

At the same time, some people know exactly how to perform, but they do not perform well, because internal self-referential factors regulate the relationship between cognition and behavior (Bandura, 1977). How individuals judge their own abilities and how this judgment affects their motivation and behavior are the most critical factors for this study (W. Zhou & Guo, 2006). Bandura (1986) developed the concept of self-efficacy through a rational grasp of human nature and its causal decision patterns, and described it as the expectation of an individual’s ability to perform a behavior in a given situation. He argued that expectations mediate cognition and behavior, and are determinants of behavior (Bandura, 1986), which means that the stronger the perceived efficacy expectations, the greater the tendency to make a greater effort (Steffen et al., 2002). In sport, self-efficacy and sport participation are reciprocally related (McAuley & Blissmer, 2000), and self-efficacy can be both a determinant (Sheeran et al., 2016) and an outcome of exercise behavior (T. J. Higgins et al., 2014). Individuals with elevated self-efficacy demonstrate higher levels of physical activity, especially when faced with challenging activities (Dishman et al., 2005). Although self-efficacy, as a consumer characteristic, is a key prerequisite for the acceptance of non-personal service technologies (Dabholkar & Bagozzi, 2002), and numerous studies have demonstrated the intrinsic relationship between sports participation and an individual’s self-efficacy (Kan & Xie, 2024; Martinez Ramirez et al., 2024; Patterson et al., 2022; Ren et al., 2020). In other words, prior studies have investigated the relationship between self-efficacy and sports participation, as well as the association between digital technology acceptance and sports engagement, there remains a paucity of research comprehensively examining how these factors collectively influence sports participation. Specifically, it is not clear whether participants’ sense of self-efficacy will influence the decision-making process regarding the use of creative and challenging new things (digital technologies) in the context of sports participation. Therefore, it is necessary to analyze the role that self-efficacy plays in the behavioral reasoning process to understand the deeper psychological mechanisms behind decision-making, which can better supplement the existing knowledge.

Therefore, this study employs the BRT to construct a theoretical model analyzing the role of self-efficacy in the reasoning process to elucidate the behavioral decision-making of sports participants utilizing digital technology for sports engagement. To achieve this research objective, the study first examines the relationship between the reasons, attitudes, and intentions for using digital technology in sports participation, and also confirms the impact of participants’ self-efficacy on the decision-making mechanisms for using digital technology in sports participation. Developers and marketers who apply these insights can formulate effective strategies to improve the experience and satisfaction of sports participants utilizing digital technology in their engagement.

## 2. Theoretical Background and Hypotheses

### 2.1. Behavioral Reasoning Theory (BRT)

BRT, introduced by Westaby (2005), is a relatively recent theory in marketing and can be regarded as an advancement of other behavioral theories, such as the Theory of Reasoned Action, the Theory of Planned Behavior, the Technology Acceptance Model, Unified theory of acceptance and use of technology and the Unified Theory of Acceptance and Use of Technology 2 (E. Jin & Lee, 2023). BRT shares some commonalities with these theories but also offers distinct advantages (Ryan & Casidy, 2018). Firstly, BRT incorporates a dual-structure model, which includes two opposing motivational factors: reasons for adoption (hereafter referred to as RFA) and reasons against adoption (hereafter referred to as RAA). These opposing perspectives are important yet contrasting views that influence users’ intentions and actual behaviors, and they offer a more thorough explanation of human decision-making phenomena by incorporating both positive and negative perspectives. Secondly, BRT highlights the critical role of values or beliefs in the reasoning process. (Sahu et al., 2020). Lastly, the BRT model offers a superior explanation of the dependent variable compared to other behavioral theories (Claudy et al., 2015).

### 2.2. Attitudes and Global Motivation

Westaby (2005) categorizes attitude (ATT), subjective norm (SN), and perceived behavioral control (PBC) as elements of global motivation, which are effective in predicting behavioral intention across a broad spectrum of behavioral research. Attitude is often considered one of the most critical predictive factors in consumer decision-making processes (Ajzen, 2001). For example, studies on the willingness of American households to adopt green energy (Wiser, 2007) and the acceptance of learning management systems (Stockless, 2018) have consistently identified attitude (ATT) as the most influential factor in shaping behavioral intention (BI). Consequently, consistent with previous studies, this research considers attitude as the sole indicator of global motivation (Qian & Kim, 2024).

### 2.3. Values and Reasons

The value of “Openness to Change (hereafter referred to as OTC)” is characterized by being open-minded, creative, open to exploring new things and accepting the associated risks (Schwartz, 1992). This finding is rooted in Rokeach’s (1973) research and specifically encompasses values such as stimulation, hedonism, and self-direction, reflecting their relevance to individual motivation and decision-making processes (Tewari et al., 2023). Currently, it is not clear how the specific perception of sports participants regarding digital technology as an innovation and its application in the field of sports affects their willingness to adopt digital technology. Therefore, this study will conduct an analysis using the concept of “OTC”.

Values are a critical structure that influences the reasons and attitudes towards the adoption and rejection of new technologies (Ashfaq et al., 2021). Individuals with a high OTC are more likely to be attracted to new products or services, showing a stronger tendency to explore novel experiences (Gupta & Arora, 2017a). For example, Sivathanu (2018a), in the context of wearable technology in healthcare, based on a survey of 815 respondents, discovered that the value of OTC positively and significantly influenced the RFA, while negatively and significantly affecting the RAA. Accordingly, we propose the following two hypotheses:

**H1a:**  *The values (OTC) of sports participants positively influence their RFA digital technology.*

**H1b:**  *The values (OTC) of sports participants negatively influence their RAA digital technology.*

### 2.4. Values and Attitudes

Values may directly influence attitudes without the mediating role of reasons (Sivathanu, 2018b), as human behavior involves a variety of unique, systematic, and complex psychological processes (Westaby, 2005). Consequently, values are also a key antecedent of attitudes (Claudy et al., 2015). This perspective is shared by scholars in the marketing field, that is, individuals with a high OTC are more likely to be attracted to new products or services, showing a stronger tendency to explore novel experiences (Gupta & Arora, 2017a; Sivathanu, 2018a), who acknowledge a close association between values and attitudes. For instance, research by Claudy et al. (2013) found that consumers are more likely to develop positive sentiments toward products or services that align with their personal values, leading to stronger preference. Building on this, we propose the following hypothesis:

**H2:**  *The values (OTC) of sports participants positively influence their attitudes toward adopting digital technology.*

### 2.5. Reasons and Attitudes and Adoption Intentions

#### 2.5.1. Reasons for Adoption (RFA)

Previous research has indicated that perceived usefulness (hereinafter referred to as PU) and perceived ease of use (hereinafter referred to as PEOU) influence attitudes and behaviors (Davis, 1989). Some scholars have discovered that PU and PEOU do not entirely explain consumers’ behavioral intentions (C.-H. Jin, 2014). This behavioral intention is driven by both extrinsic motivations (usefulness) and intrinsic motivations (enjoyment) (R. Zhou & Feng, 2017). Additionally, PU reflects utilitarian motivations for using technology, PEOU is related to effort expectancy, while perceived enjoyment (hereinafter referred to as PE) represents hedonic motivations (Huang & Ren, 2020). Therefore, this study will use PU, PEOU, and PE as key factors in the adoption of digital technology for sports participation.

PEOU and PU play a crucial role in explaining an individual’s behavior when adopting new technologies (Rauniar et al., 2014). These perceptions are significant predictors that foster a positive attitude towards technology use, a finding that has been consistently supported across various studies (Ko et al., 2011; Alharbi & Drew, 2014; Amin et al., 2017). This includes Taekwondo scoring techniques, college students’ adoption of e-learning systems, and teachers’ use of learning management systems. PE, as a hedonic motivation, can affect customer satisfaction and influence the intention to continue using a service (Akdim et al., 2022). Both Hew et al. (2018) and Akdim et al. (2022) have found that when users experience pleasure in using mobile applications, they are more likely to continue using them. Therefore, the following hypotheses are proposed to explore these relationships further:

**H3a:**  *The RFA digital technology will positively influence sports participants’ attitudes toward using digital technology.*

**H4a:**  *The RFA digital technology will positively influence sports participants’ intention to use digital technology.*

#### 2.5.2. Reasons Against Adoption (RAA)

The Diffusion of Innovations model primarily examines the factors that promote the adoption of innovations and pays less attention to the resistance people may have towards new technologies and innovations. However, resistance serves as a significant limiting factor in the acceptance of innovations (P.-T. Chen & Kuo, 2017). Consequently, scholars have developed the Theory of Innovation Resistance to discuss the factors that impede the adoption of innovations (Ram & Sheth, 1989). In their pioneering work, Ram and Sheth identified functional and psychological barriers as two main types of resistance factors, which are further categorized into RAA, such as usage barriers (hereinafter referred to as UB), value barriers (hereinafter referred to as VB), risk barriers (hereinafter referred to as RB), traditional barriers (hereinafter referred to as TB), and image barriers (hereinafter referred to as IB). These factors have been found to hinder the spread of innovations in various studies. For instance, the development of electric vehicles (EVs) may be constrained by UB (Zhang et al., 2011). Laukkanen (2016) found that VB are the strongest inhibitors of the adoption and intention to use m-banking services, while RB may create a sense of insecurity among users of online travel agencies, thereby suppressing their intention to use these services (Talwar et al., 2020).

TB are also a cause of consumer resistance to mobile commerce applications (Hew et al., 2023), and negative image perceptions can ultimately lead to the rejection of innovations (Kleijnen et al., 2009). In the sports domain, Uhrich (2022) investigated the impact of fan resistance to experiential applications specifically driven by declining atmosphere (UB), distraction from the game (TB), and perceived social risks coupled with data security concerns (RB)—on usage attitudes and adoption intentions. Although other studies on innovation resistance offer different perspectives, they generally align with the aforementioned barriers to innovation. Therefore, this study employs five factors—UB, VB, TB, RB, and IB—as lower-order dimensions representing the RAA, and proposes the following hypotheses:

**H3b:**  *The RAA digital technology will have a negative influence on sports participants’ attitudes towards using digital technology.*

**H4b:**  *The RAA digital technology will have a negative influence on the adoption intentions of sports participants to use digital technology.*

### 2.6. Attitudes and Adoption Intentions

Eagly and Chaiken (1993) define attitude as “a psychological tendency that is expressed by evaluating a particular entity with some degree of favor or disfavor”. Consumer intentions are considered a distinct psychological cognition from attitudes (Honkanen et al., 2006), yet individuals who hold a more positive attitude toward innovation are more likely to adopt it (Bagozzi, 1992). It is crucial to understand the general consumer’s acceptance of technology or innovation because technology has no value unless it is accepted and utilized (Oye et al., 2014). Numerous studies (Claudy et al., 2015; Gupta & Arora, 2017a, 2017b) have confirmed that a positive attitude significantly influences behavioral intentions, such as the adoption of m-banking, m-shopping, and innovation adoption in general. In various domains (Anayat et al., 2023; Jan et al., 2023; Kasilingam, 2020; Kottasz et al., 2021; McLean & Osei-Frimpong, 2019; Verma et al., 2019), consumer attitudes have been found to positively affect their intentions to use corresponding technologies, including mobile technology, green consumption, intelligent chatbots, AI customer service, AI voice assistants, and autonomous vehicles. Although the use and potential of digital technology have been extensively discussed in the literature, studies analyzing consumer decision-making processes regarding the use of digital technology through the Behavioral Reasoning Theory are limited. Thus, the hypothesis is proposed as follows:

**H5:**  *The attitudes of sports participants towards using digital technology in sports participation will have a positive influence on their intentions to use digital technology.*

### 2.7. Values and Adoption Intentions

The role of OTC in prior behavioral research has been extensively examined. For instance, investigations into online travel agency (OTA) adoption (Hien et al., 2024) demonstrate that consumers with stronger OTC orientations exhibit greater propensity to leverage OTA benefits, thereby mitigating perceived drawbacks associated with service adoption, and enhancing usage intentions. Similarly, in the sports domain, Skimina et al. (2021) identified a positive correlation between OTC and physical activity engagement. Furthermore, individuals with elevated OTC levels demonstrate increased likelihood of adopting fitness applications, with application-generated behavioral data enabling granular categorization of user behaviors and facilitating exercise optimization (Mejova & Kalimeri, 2019). In other words, fostering a more proactive and positive attitude toward innovative products and a greater willingness to adopt them (Tewari et al., 2022). The high integration of digital technology with sports has resulted in the emergence of new technologies or innovations. This raises the question of whether participants’ values (i.e., a preference for novelty and new experiences) influence their inclination to adopt such innovations. Thus, we propose following hypothesis:

**H6:**  *The values (OTC) will have a positive influence on sports participants’ adoption intentions to use digital technology.*

### 2.8. The Moderating Role of Self-Efficacy

Self-efficacy, described as the expectation of whether an individual has the capability to execute actions in specific situations, is extremely important (Bandura, 1986). This expectation serves as a mediator between cognition and behavior and is a decisive factor in behavior. Self-efficacy can assess an individual’s own capabilities and how this assessment influences their motivation and behavior (W. Zhou & Guo, 2006). This implies that the stronger the perceived efficacy expectation, the more inclined an individual is to exert greater effort (Steffen et al., 2002). Self-efficacy influences individual behavior across four key aspects: behavioral choice, motivational effort, cognitive processes, and emotional processes, shaping how individuals act, persevere, think, and respond emotionally in various situations (W. Zhou & Guo, 2006). The BRT suggests that reasons play a rational role in shaping global motivation and intentions. These reasons not only guide individuals in forming intentions but also serve to justify and defend their actions, ultimately preserving and enhancing their sense of self-worth (Westaby, 2005). BRT also assumes that when new information challenges an individual’s self-perception, it may interrupt ongoing pursuits, serving as an important factor in assessing one’s capabilities and influencing behavior and motivation, consistent with the role of self-efficacy in the decision-making process of individual behavior. Previous studies have largely explored the role of self-efficacy within the Theory of Planned Behavior (Alam et al., 2023; Doanh, 2021; Shi et al., 2017), analyzing its moderating effects between attitudes, subjective norms, perceived behavioral control, and intentions. The influence of self-efficacy in the framework of BRT remains unexplored, leaving a gap in understanding its role in shaping reasoning and behavior. Therefore, we propose the following hypotheses:

**H7a:**  *The relationship between values of OTC and RFA is moderated by self-efficacy.*

**H7b:**  *The relationship between values of OTC and RAA is moderated by self-efficacy.*

**H7c:**  *The relationship between values of OTC and attitudes is moderated by self-efficacy.*

**H7d:**  *The relationship between RFA and attitudes is moderated by self-efficacy.*

**H7e:**  *The relationship between RAA and attitudes is moderated by self-efficacy.*

**H7f:**  *The relationship between RFA and adoption intentions is moderated by self-efficacy.*

**H7g:**  *The relationship between RAA and adoption intentions is moderated by self-efficacy.*

**H7h:**  *The relationship between attitudes and adoption intentions is moderated by self-efficacy.*

**H7i:**  *The relationship between values of OTC and adoption intentions is moderated by self-efficacy.*

**Figure 1:**
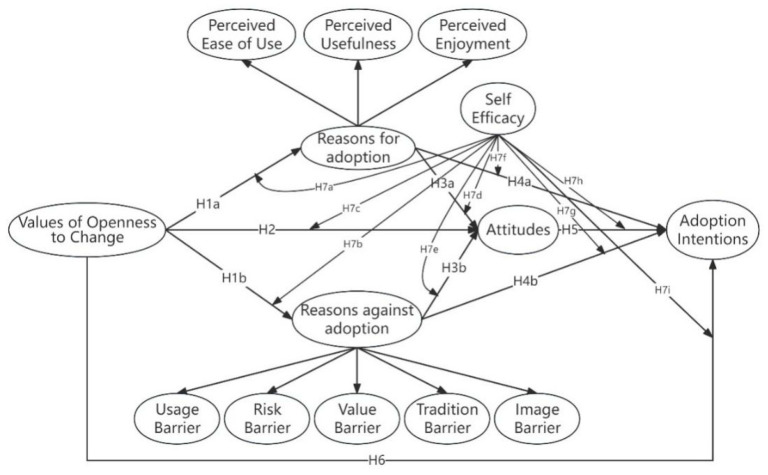
Research model.

The theoretical model is shown in Figure 1.

## 3. Methodology

### 3.1. Subjects of the Study

The target population for this study comprises individuals residing in China who engage in sports activities facilitated by digital technology, focusing on their behaviors and motivations. Since it was not feasible to randomly sample sports participants utilizing digital technology from the general population, data were collected through an online survey using a convenience sampling approach, which was regarded as an effective and time-efficient strategy to gather relevant responses from the target group. During the Hangzhou Asian Games, a web-based questionnaire was distributed via social networking platforms such as WeChat, Weibo, Bilibili, and TikTok. The Hangzhou Asian Games showcased various digital technologies in sports, providing an opportune moment to collect responses from the target population, which is reasonable for the conduct of the study. Specifically, during the sporting events, these social media platforms disseminated news coverage highlighting the application of digital technologies—including artificial intelligence (AI), intelligent interaction, and virtual reality—during the Asian Games. Crucially, these technologies were reported to enhance the athletic engagement of participants. Subsequently, individuals who commented on or expressed approval (via “likes”) in the comment sections of these news posts were contacted via direct messaging and invited to participate in the survey. This approach ensured that respondents possessed a foundational understanding of and interest in the integration of digital technologies within the sports domain. The questionnaire initially surveyed the frequency of using digital technology for sports participation, and a self-assessment of frequency of use was conducted, yielding a total of 417 questionnaires. Subsequently, questionnaires that were not answered seriously were eliminated following the method of Zhong et al. (2021), resulting in 318 valid questionnaires, as shown in Table 1. Among them, males (55.3%) outnumbered females (44.7%), with the age group of 31–40 years old being the most prevalent, accounting for 32.1%. Respondents with a bachelor’s degree (49.1%) were the largest group, and the majority of the group (33.3%) had an income below 2000 yuan. The demographic information is detailed in Table 1.

To roughly assess the extent to which the sample of this study approximates the population in China that engages in sports using digital technology, the study utilized the following objective and recent demographic variables: First, according to the “2020 China Population Census Yearbook”, the gender ratio is 51.17% male and 48.83% female. Additionally, a survey on the digital literacy level of Chinese residents by M. Chen and Chen (2024) found that males have higher levels of both basic and advanced digital literacy compared to females. In this study, which is based on an online survey, the gender distribution is 55.3% male and 44.7% female, which is roughly in line with the population characteristics. Secondly, regarding the frequency of using digital technology during sports participation, 67.3% of respondents reported usage below 50%, which aligns with the findings by M. Chen and Chen (2024) that a significant portion of Chinese residents have a basic level of digital literacy. Furthermore, a report published by the National Physical Fitness Monitoring Center (2020) indicates that the proportions of children, adults, and the elderly who participate in physical exercise at least once a week are 35.8%, 29.7%, and 5.8%, respectively, showing a trend of decreased exercise with increasing age. According to the latest statistical survey on the development of the Internet in China by the China Internet Network Information Center (2024), the proportions of citizens aged 10–19, 20–29, 30–39, 40–49, and 50 and above are 13.6%, 13.5%, 19.3%, 16.7%, and 33.3%, respectively. Considering both sports participation and internet usage, this suggests that the survey sample of this study is representative of the population characteristics. Overall, the above arguments seem to indicate that the sample of this study, to some extent, reflects the population in China that engages in sports using digital technology.

### 3.2. Survey Procedure

Researchers focused on groups actively engaging on social networking platforms during the Hangzhou Asian Games, such as those participating in discussions, liking, or saving videos and news announcements. These include content that combines sports with digital technology (e.g., stadium VR experiences, intelligent sports, intelligent assessments, etc.). Subsequently, the respondents will be provided with a detailed explanation of the research objectives, procedures and methods, the time required, and the confidentiality of the data collected, and will be asked whether they are willing to participate in the survey. Only those who agreed to be surveyed were given the questionnaire to ensure their informed consent and ethical protection of the respondents. To ensure the survey’s effectiveness, respondents’ willingness to participate in sports was first assessed, including their interest in physical exercise, game viewing, or specific sports-related consumption. At the initial stage of the questionnaire, a detailed introduction to the applications and functions of digital technology in sports was provided to ensure that respondents understood its relevance and were able to complete the survey independently. Ultimately, 417 questionnaires were obtained, and then questionnaires that were not answered seriously were eliminated following the method of Zhong et al. (2021), resulting in 318 valid questionnaires.

### 3.3. Measurement Tools

The measurement tool used in this study is a questionnaire that consists of latent variables such as the values of OTC, RAA, RFA, attitudes (ATT), and adoption intentions (AI), as well as questions related to demographic characteristics. The values of OTC, UB, and IB were developed by referring to the study by Pillai and Sivathanu (2018). RB are integrated from the studies of Gupta and Arora (2017a) and Sivathanu (2018b) and modified for this study. TB were adapted from previous studies (Pillai & Sivathanu, 2018; Sadiq et al., 2021; Sivathanu, 2018a). PEOU and PU are adapted from the research of Alharbi and Drew (2014). PE is adapted from the study of Thong et al. (2006). ATT are adapted from the research of Claudy et al. (2015) and Pillai and Sivathanu (2018). AI is adapted from the study of Fishbein and Ajzen (1975). Self-efficacy (SE) is adapted from the research of Schwarzer et al. (1997). The specific measurement items are detailed in Table 2. All the observed variables in this study are assessed on a seven-point Likert scale.

### 3.4. Data Analysis Methods

This study employs SPSS 27 and SmartPLS 4.1 for data analysis, utilizing SmartPLS to analyze the structural equation modeling due to its several advantages (J. F. Hair et al., 2022). Firstly, Partial Least Squares Structural Equation Modeling (hereinafter referred to as PLS-SEM) can handle and validate models with complex relationships (Chin et al., 2020); secondly, PLS-SEM serves as a “causal predictive” technique with strong predictive capabilities (Anayat et al., 2023); and lastly, PLS-SEM is particularly well-suited for both explanatory and exploratory research, as it effectively models complex relationships and helps uncover underlying patterns (J. F. Hair et al., 2022), making it a reasonable choice for analysis and validation in this study. Based on this, the study uses SPSS 27 for descriptive statistics of the sample. Subsequently, the reliability and validity of the measurement model are rigorously tested to ensure that the survey results possess adequate reliability and stability. This also ensures the internal consistency of the variables and that there is enough distinction between variables to truly reflect their characteristics. After validating the robustness of the model with Smart PLS 4.1, all hypotheses of the structural model are tested.

### 3.5. Assessment of the Measurement Model

Based on previous research and hypotheses, this study constructs a theoretical model, which follows a reflective–reflective type, with “RFA” and “RAA” modeled as higher-order constructs. Therefore, a two-stage approach is employed to measure the higher-order structures within the structural equation model (J. F. Hair et al., 2022). First, only the lower-order constructs within the measurement model are assessed. This is performed through PLS-SEM analysis and evaluation to obtain the latent variable scores. The derived latent variable scores are then saved and reintroduced for further analysis in the second stage. In the second stage, these latent variable scores are employed to assess the higher-order constructs. The two-stage method provides a flexible, robust, and easily implementable approach that helps to better handle complex data structures and model relationships, thereby enhancing the quality and reliability of the results (Sarstedt et al., 2019). Next, we assessed the overall quality (reliability and validity) of the questionnaire. Convergent validity was evaluated through factor loadings, Cronbach’s alpha, Variance Inflation Factor (hereinafter referred to as VIF), Average Variance Extracted (hereinafter referred to as AVE), and Composite Reliability (hereinafter referred to as CR). Discriminant validity was evaluated by applying the Fornell and Larcker criterion along with the Heterotrait–Monotrait Ratio of Correlations (hereinafter referred to as HTMT) method to ensure distinctiveness between constructs. Finally, this study conducted a robustness check to ensure the reliability of the results. The specific results can be found in Table 2 and Table 3. 

To ensure the reliability of the results, this study first employs Variance Inflation Factor (VIF) analysis to examine potential collinearity issues among variables, ensuring that the survey data meet the basic assumptions required for multiple regression analysis (Kock, 2015). As indicated in Table 2, all variables’ VIF values are below the recommended threshold, indicating no multicollinearity issues (J. F. Hair et al., 2022). According to the results, the minimum Cronbach’s Alpha coefficient is 0.708, meeting the commonly accepted threshold of 0.7 or higher for internal consistency as proposed by previous scholars (Bagozzi & Yi, 1988). All observed variables have statistically significant factor loadings (*p* < 0.001), with a minimum of 0.763, exceeding the threshold of 0.50. This is consistent with the recommendations of Nunnally and Bernstein (1994), which suggest that factor loadings greater than 0.5 indicate satisfactory validity. Building on this foundation, the study further analyzes CR, AVE, the Fornell and Larcker criterion, and HTMT to validate the convergent and discriminant validity of the measurement instrument.

To ensure that the dataset for the study variables exhibits good convergent and discriminant validity, validity analysis results are obtained by calculating CR, AVE, the Fornell and Larcker criterion, and HTMT. J. Hair et al. (2014) suggest that an AVE greater than 0.5 and a CR greater than 0.7 indicate satisfactory levels of convergent validity, ensuring the constructs’ consistency and measurement accuracy. Additionally, the square root of AVE for each measurement construct should be greater than the estimated inter-construct correlations (Fornell & Larcker, 1981), and all HTMT values fall within the acceptable range, that is, below the threshold of 0.85 (Henseler et al., 2015). Therefore, the measurement model has satisfactory discriminant validity. Finally, the model’s fit indices were assessed, revealing an SRMR of 0.075, which is below the threshold and meets the requirements of the study (J. F. Hair et al., 2022).

Building on the established convergent and discriminant validity, the robustness of the research model is further analyzed through heterogeneity tests (Finite Mixture Segmentation, FIMIX), endogeneity tests, and nonlinearity checks (J. F. Hair et al., 2022). The results indicate the absence of endogeneity and nonlinearity issues, which is crucial for the reliability of the model’s estimates. In the heterogeneity test, potential segments were set at 2, 3, and 4 (further segmentation was terminated when set to 5 due to insufficient sample size in the resulting groups). The normed entropy statistic (EN) was found to be greater than 0.5 in all cases, suggesting the potential presence of heterogeneity. Subsequent analysis using multiple group analysis for all potential segments revealed no differences between the groups, indicating that there is no latent heterogeneity. This implies that the model has good robustness (Sarstedt et al., 2020). Finally, the structural model is assessed using path coefficients, t-values, and the explained variance (R^2^). A non-parametric bootstrapping procedure (5000 resamples) is employed to test the significance of the path coefficients (Davison & Hinkley, 1997).

## 4. Results

### 4.1. Structural Model Assessment

This study employed PLS-SEM for path analysis to test all hypothesized relationships. The specific results, as shown in Table 4, indicate the following: The values of the respondents positively influence the RFA (β = 0.223, *p* < 0.001) and negatively impact the RAA (β = −0.217, *p* < 0.001), thus confirming Hypotheses H1a and H1b. Values of OTC positively impact their attitudes (β = 0.287, *p* < 0.001), which means Hypothesis H2 is supported. The RFA have a positive impact on attitudes (β = 0.148, *p* < 0.01), while the RAA have a negative impact on attitudes (β = −0.296, *p* < 0.001), thus confirming Hypotheses H3a and H3b. The RFA positively impact the adoption intentions (β = 0.14, *p* < 0.01), and the RAA negatively impact the adoption intentions (β = −0.136, *p* < 0.01), thus confirming Hypotheses H4a and H4b. The attitudes positively impact the adoption intentions (β = 0.23, *p* < 0.001), which leads to the support of Hypothesis H5. Values of OTC positively impact the adoption intentions (β = 0.296, *p* < 0.001), thus supporting Hypothesis H6.

This study uses three indicators (R^2^, Q^2^, f^2^) to evaluate the structural model. The R^2^ values indicate the proportion of variance explained by the model, with thresholds of 0.25 for weak, 0.50 for moderate, and 0.75 for strong explanatory power. The specific results are presented in Table 5, indicating a moderate level of explanatory power. Furthermore, the Q^2^ value is 0.335, suggesting that the proposed framework has moderate predictive relevance (J. F. Hair et al., 2022). Lastly, f^2^ is evaluated based on thresholds established by Cohen (1988), f^2^ ≥ 0.02 (small effect sizes), f^2^ ≥ 0.15 (medium effect sizes), and f^2^ ≥ 0.35 (large effect sizes). As shown in Table 5, for all significant path relationships, the exogenous variables have a substantial influence on the endogenous variables, which is an important indicator of the practical significance of the model.

The second-order coefficients from the study provide insight into the relative importance of different factors influencing the RFA and RAA of digital technology in sports participation. For the RAA, the results indicate that usage barriers (β = 0.838, *p* < 0.001) are the most significant factor, followed by value barriers (β = 0.825, *p* < 0.001), traditional barriers (β = 0.794, *p* < 0.001), image barriers (β = 0.763, *p* < 0.001), and risk barriers (β = 0.751, *p* < 0.001). These results highlight the key concerns that may lead individuals to resist the adoption of digital technology in sports. On the other hand, the second-order coefficients for the RFA reveal that perceived enjoyment (β = 0.807, *p* < 0.001) is the most influential factor for sports participants to accept the use of digital technology, suggesting that the pleasure and interest derived from the technology play a crucial role in adoption. Perceived ease of use (β = 0.792, *p* < 0.001) and perceived usefulness (β = 0.784, *p* < 0.001) are also identified as important factors, indicating that how easy the technology is to use and the perceived benefits of using it significantly contribute to the acceptance decision.

### 4.2. Moderation Analysis

**Figure 2:**
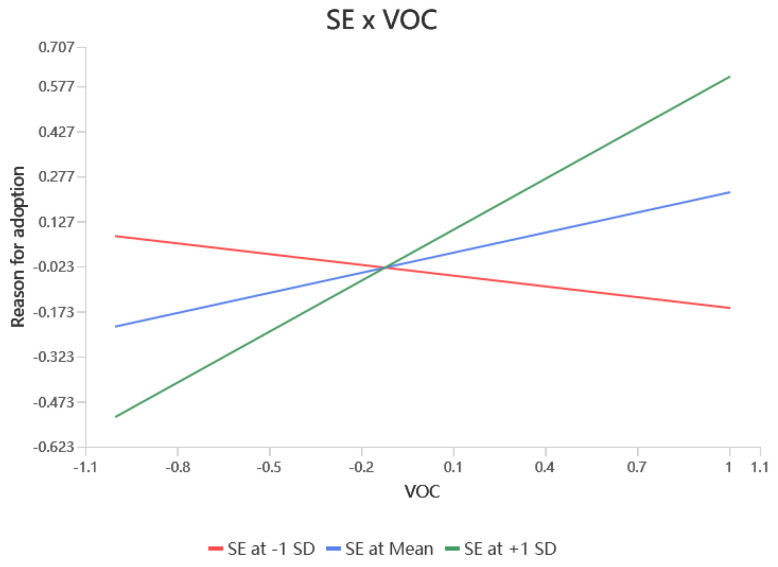
Interaction slope.

**Figure 3:**
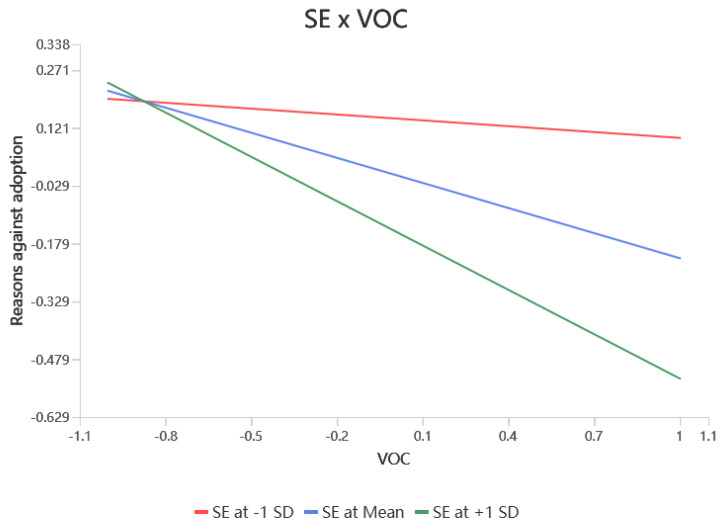
Interaction slope.

**Figure 4:**
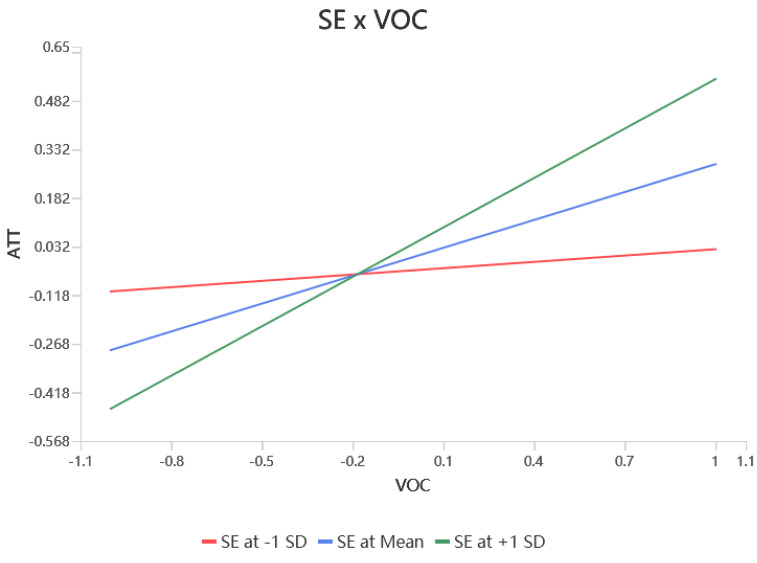
Interaction slope.

**Figure 5:**
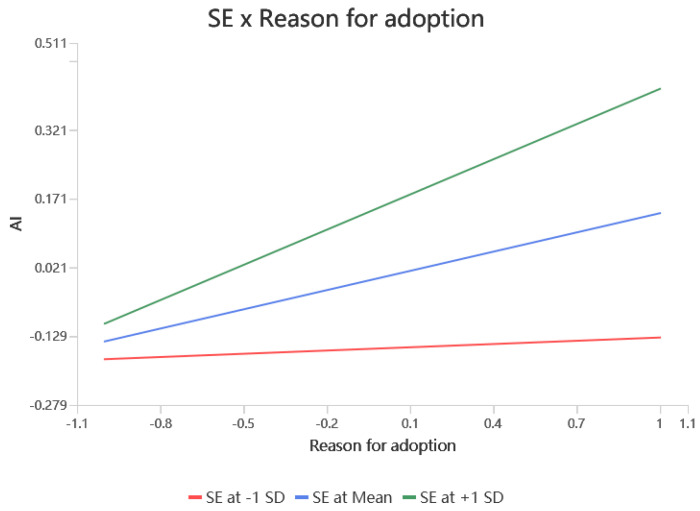
Interaction slope.

**Figure 6:**
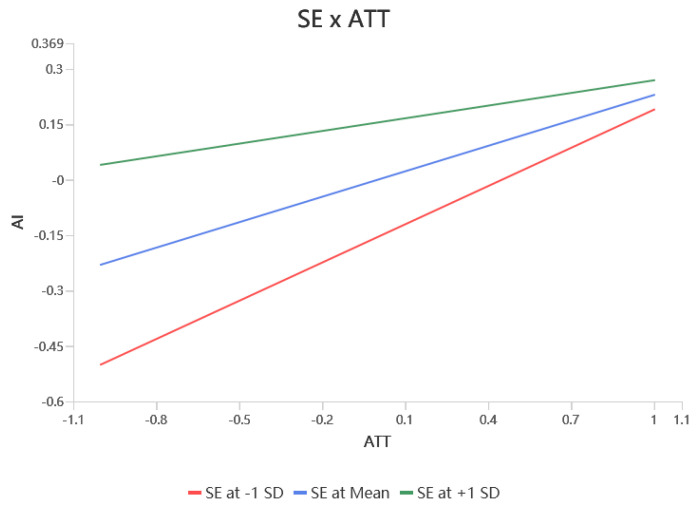
Interaction slope.

According to Table 4, self-efficacy significantly moderated the relationship between values and RFA (β = 0.342, *p* < 0.001), RAA (β = 0.166, *p* < 0.01), and attitudes (β = 0.222, *p* < 0.001), resulting in hypotheses H7a, H7b, and H7c being supported. Self-efficacy did not significantly modulate the relationship between RFA (β = 0.095, *p* > 0.05) and RAA (β = 0.049, *p* > 0.05) on attitudes, i.e., hypotheses H7d and H7e were not supported. Self-efficacy would have significantly moderated the relationship between RFA (β = 0.116, *p* < 0.05) and attitudes (β = −0.115, *p* < 0.01) on the adoption intention, resulting in support for hypotheses H7f, and H7h. Finally, self-efficacy did not significantly moderate the effects of RAA (β = −0.019, *p* > 0.05) and attitudes (β = 0.038, *p* > 0.05) on adoption intentions, leaving hypotheses H7g, H7i unsupported. The simple slopes of the significant moderating effects are shown in Figure 2, Figure 3, Figure 4, Figure 5 and Figure 6.

## 5. Discussion

### 5.1. Theoretical Implications

Firstly, this study develops and tests a model using Behavioral Reasoning Theory (BRT) and the theory of resistance to innovation, analyzing the behavioral decision-making process by considering both RFA and RAA. This represents an important theoretical extension of the existing innovation framework for sports consumers’ behavioral decision-making. Consistent with previous study findings (Sivathanu, 2018a; Pillai & Sivathanu, 2018; Qian & Kim, 2024), the RFA and RAA significantly impact sports participants’ attitudes and intentions to use digital technologies (positive for adoption and negative against adoption). This suggests that sports participants’ psychological and behavioral responses are influenced by factors of resistance, ultimately affecting the adoption of digital technology for sports participation. That is, the two opposing sets of motivations jointly affect the attitude and behavioral decision to use digital technology for sports participation. Simultaneously, this study reveals the relative influence of different reasons on the results, indicating that the attitude and behavior of adoption digital technology are more affected by RAA than by RFA. This is consistent with the finding that sports consumers tend to be reticent about technological innovations (Naraine, 2019). At the same time, Ehnold et al. (2023) identified four distinct digital adoption archetypes—”skeptics”, “enthusiasts”, “pessimists”, and “high achievers”—through empirical clustering analysis. Notably, pessimists and a subset of skeptics exhibited heightened perceptions of digital risks (e.g., privacy breaches and technological overreliance), suggesting that these attitudinal profiles may constitute psychological barriers to sports consumers’ acceptance of technological innovations. This study analyzes the specific elements of RFA (PU, PEOU, PE) and RAA (UB, IB, RB, VB, TB) and investigates the relative relationships between each element and the higher-order reasons. Perceived enjoyment was found to have the strongest correlation with the overall RFA, which aligns with the findings of Uhrich (2022). This may suggest that sports consumers are more likely to adopt innovations during the consumption process due to the hedonic elements involved. On the other hand, barriers to use were found to be the most strongly correlated factor for the overall RAA. Consumers readily accept new innovations when they perceive that they do not require much effort (Msaed et al., 2017). That is, usage barriers are the primary factor contributing to rejection, significantly constraining the behavioral intentions of sports participants (Qian & Kim, 2024). In addition, this study explored the role of values in specific behavioral decisions, significantly influencing RFA, RAA and attitudes, a finding that aligns with prior studies (Gupta & Arora, 2017b; Sivathanu, 2018a). Since values play a foundational role in behavioral decision-making (Westaby, 2005), they simultaneously affect consumer attitudes and eventual adoption intentions. In other words, considering participants’ perceptions of new technologies is a critical factor that should be prioritized in the process of designing and developing related products.

Another important finding was the clarification of the specific role of participants’ self-efficacy in behavioral decision-making about using digital technologies for sport participation. Firstly, self-efficacy was found to positively and significantly modulate the relationship between values and RFA and attitudes. This implies that individuals with high levels of self-efficacy have a greater influence on their values regarding the acceptance of adoption reasons and attitudes. Values are antecedents to behavioral decision-making (Westaby, 2005), and reasons are the motivations that individuals use to rationalize their actions and justify their behaviors, thereby defending their own values. Self-efficacy is an important factor for individuals to control their behaviors and meet their expectations (W. Zhou & Guo, 2006). This psychological factor helps individuals to justify and defend their behaviors, effectively explaining the causal relationship between the significant reinforcement of individuals’ values of OTC influencing the adoption of rational acceptance and attitudes. The relationship between the values of OTC and RAA is positively influenced by self-efficacy. Furthermore, the relationship between the values of RFA and intentions to adopt is positively influenced by self-efficacy, as reason can be the most powerful drivers of behavioral intentions (Davis et al., 1989). However, it was surprising that the relationship between RAA and intention to adopt was not moderated by self-efficacy. This could be due to the intrinsic motivation and value assessment of consumers, consistent with the idea that consumers tend to be reticent towards technological innovation (Naraine, 2019). Barriers are a significant factor in influencing sports participants’ resistance to innovation, and they do not differ based on the strength of participants’ self-efficacy. Furthermore, given the presence of “Doubters” and “Pessimists” (Ehnold et al., 2023) self-efficacy may not significantly moderate the relationship between the RAA and behavioral intentions when the core antecedents of RAA—such as privacy concerns, prohibitive costs, and preference for traditional methods—are orthogonal to individual capability. The relationship between attitudes and adoption intentions is positively influenced by self-efficacy. An interesting finding is that this relationship is significantly weakened, implying that the group with stronger self-efficacy will be less willing to use digital technology. This aligns with other similar studies that have produced different results (Shah Alam et al., 2023). Individuals with higher self-efficacy may be less interested in using digital technology for sports participation because a more comprehensive comparison is necessary. The ability to manipulate behavioral expectations is extremely important (Bandura, 1986), and expectations are determinants of behavior. Other factors, such as perceived benefits or disadvantages, familiarity with the technology, ease of use, and the direct effect of the technology on enhancing sports performance, may more directly influence adoption intentions. Overall, the more positive the attitude towards using digital technology for sports, the greater the likelihood of using digital technology for future sports participation. However, groups with higher self-efficacy had a relatively weaker influence on attitudes towards digital technology, whereas groups with lower self-efficacy were more influenced by their own attitudes. This implies that groups with lower self-efficacy need to develop positive attitudes towards digital technology when engaging in physical activities that use digital technology. Finally, the study found that self-efficacy does not have a significant impact on the relationship between personal values of OTC and adoption intentions. OTC is a value that motivates people to pursue interests in unforeseen and uncertain directions (Schwartz, 1992), characterized by open-mindedness, creativity, and a willingness to try new things and take risks (Kruse et al., 2018). This implies that sports participants care more about the individual’s attempts to try new things, and with values being a precursor to behavior, it is reasonable that the self-efficacy of individual participants does not significantly influence the relationship between values of OTC and the willingness to use digital technology for sports participation.

### 5.2. Practical Implications

Digital technology acts as a potential new source of momentum in the sports domain, which is extremely important for sports participants and sports-related industries. For sports participants, digital technology can assist in their engagement in sports and help them to achieve their set goals. For related industries, the application of digital technology can address underlying challenges within the sports sector, thereby propelling industry growth. Thus, this study offers novel perspectives for identifying specific views and analyzing consumer behavior decisions. Firstly, the RFA and RAA of digital technology both influence decision-making in sports participation, with perceived enjoyment and usage barriers being particularly pivotal in affecting sports participants’ decisions to adopt or resist digital technology for sports involvement. This implies that developers of digital technology should have a clear understanding of the advantages of product use and the factors influencing resistance from the sports consumers’ viewpoint during product design and development. For instance, by enhancing the enjoyable aspects and ensuring the creation of engaging and innovative products, while also reducing usage complexity to make technology more accessible, developers can significantly promote the acceptance of digital technology and participation in sports. Moreover, it is crucial to design products that resonate with the audience’s values, particularly those who exhibit OTC, as values are foundational to behavior.

On the other hand, the study found that for sports participants with a high sense of self-efficacy, the impact of their values on the RFA and attitudes is more significant. This suggests that developers and marketers can design more promotional strategies that highlight the positive role of digital technology in sports activities. For example, they can provide more challenging digital technology application scenarios, more interactive sports training platforms, or devices with real-time feedback features to help individuals with high self-efficacy better perceive the advantages of digital technology, thereby encouraging their active adoption. For groups with low levels of self-efficacy, it is necessary to improve their understanding of digital technology through promotional activities and education and actively guide their positive attitudes towards digital technology. This can be achieved by designing simple and user-friendly technology interfaces, providing beginner’s guides or introductory training courses, or by enhancing the confidence of low-efficacy participants through community sharing and user experience stories, gradually increasing their willingness to adopt. At the same time, in practice, it is important to focus on potential obstacles of digital technology itself, such as technical complexity, cost of use, or privacy issues. These obstacles should be addressed by optimizing technical design and improving user experience to reduce their negative impact on user intentions, and targeted improvements should be made based on user feedback mechanisms for known reasons for rejection, in order to attract more potential users. Additionally, groups with a strong sense of self-efficacy tend to comprehensively assess the pros and cons before using digital technology, indicating that related digital technology products should provide detailed information, explanations of technological advantages, and trial opportunities to help high self-efficacy users make more favorable judgments. Finally, regarding the phenomenon where the relationship between values of OTC and adoption intentions is not moderated by self-efficacy, practice should place greater emphasis on the needs of sports participants for innovation and a spirit of adventure. This can be achieved by holding technology experience events or innovative competitions to stimulate users’ curiosity and enthusiasm for trying new technologies, thereby increasing their acceptance of digital technology.

## 6. Conclusions

Digital technology, as a potential new driving force for solving economic issues, has also greatly transformed the way sports participants engage in sports activities. How to leverage digital technology to promote sports participation and improve people’s overall health levels is an important focus of attention in sports-related fields. However, focusing on the needs of sports participants is key to the research and development, application, and promotion of digital technology in the field of sports. Considering the overall views of sports participants on digital technology regarding different (positive and negative) factors and clarifying the participants’ behavioral decision-making process is essential. Therefore, this study utilizes the Behavioral Reasoning Theory and finds that different RFA and RAA both have significant impacts on sports participants’ attitudes and intentions, with the impact of RAA being higher than that of RFA. Hence, the primary focus should be on reducing the inconveniences participants experience when using digital technology for sports participation. At the same time, perceived enjoyment, as an important influencing factor, should be widely concerned. Furthermore, to increase the willingness of sports participants to accept and engage with digital technology, designing products that align with consumer values is more important, as values are precursors to behavior, making related technology products more readily accepted by the relevant groups. Lastly, it is also necessary to start with the participants’ judgment of the individual, to clearly analyze how participants judge their own capabilities and how this judgment affects their willingness to use digital technology. Actively enhancing the judgment of oneself, and making the perceived efficacy expectation stronger, can thus increase the willingness of sports participants to accept digital technology and actually participate. Furthermore, differentiated design strategies should be tailored to users with varying levels of self-efficacy. For individuals with high self-efficacy, who tend to conduct comprehensive cost–benefit analyses prior to technology adoption, products should provide exhaustive informational support. These should include detailed explanations of advantages, comparative analyses, and trial opportunities, enabling informed and proactive decision-making. Concurrently, developing more challenging and interactive application scenarios—such as real-time feedback training platforms or advanced data analytics tools—can better align with high-efficacy users’ demands for technological depth, thereby reinforcing adoption intentions. For users with low self-efficacy, interventions should target cognitive enhancement and attitudinal guidance. First, awareness campaigns and educational programs can elevate their understanding of digital technologies. Second, simplifying technical design—via user-friendly interfaces, step-by-step tutorials, or peer success stories within user communities—is critical to reducing barriers and boosting confidence, thereby increasing the willingness of sports participants to accept digital technology and engage in sport.

## 7. Research Limitations and Future Outlook

This study, while offering significant insights, has certain limitations that could be addressed in future research endeavors. Firstly, due to a variety of factors such as economic, social, and cultural influences, a primary limitation of this study is that it does not account for potential differences in other regions around the world. There is a clear need to broaden the scope of the research to include a more diverse group of participants to understand how regional or cultural disparities might impact consumer decision-making processes in the context of sports participation. Secondly, in light of the rapid pace of technological evolution and societal changes, it would be beneficial for future studies to utilize longitudinal empirical designs. Such designs could track changes in participants’ behavioral decisions over an extended period, offering a more reliable theoretical basis for anticipating future trends. Thirdly, while the current study has centered on the intention to adopt digital technology, subsequent research efforts might benefit from focusing on actual user behaviors. This shift in focus would assist researchers in identifying any discrepancies between the stated intentions of participants and their real-world actions. Furthermore, this study utilized convenience sampling, which may introduce sampling bias (e.g., infrastructure convenience). Future research should refine sampling strategies and employ advanced data processing techniques to mitigate these biases. Finally, this study does not specifically investigate the individual factors of RFA and RAA. Future research should consider employing mixed research methods to uncover and analyze these underlying reasons.

## Figures and Tables

**Table 1:** Respondents’ profile.

Characteristic		Frequency	%
Gender	Male	176	55.3%
	Female	142	44.7%
Age (years old)	Under 18	37	11.6%
	18–30	85	26.7%
	31–40	102	32.1%
	41–50	63	19.8%
	51–60	17	5.3%
	Over 60	14	4.4%
Education	Middle school and below	17	5.3%
	High school	44	13.8%
	College	87	27.4%
	Undergraduate	156	49.1%
	Graduate students and above	14	4.4%
Occupation	Student	82	25.8%
	Teacher	29	9.1%
	Full-time housewife	34	10.7%
	Company employees	93	29.2%
	Freelance work	54	17.0%
	Other	26	8.2%
Income (yuan)	2000 or less	106	33.3%
	2001–4999	73	23.0%
	5000–7999	63	19.8%
	8000–9999	34	10.7%
	10,000–14,999	26	8.2%
	15,000 and above	16	5.0%
Frequency of use	10%	30	9.4%
	20%	31	9.7%
	30%	35	11.0%
	40%	54	17.0%
	50%	64	20.1%
	60%	35	11.0%
	70%	28	8.8%
	80%	25	7.9%
	90%	14	4.4%
	100%	2	0.6%

**Table 2:** Reliability and validity.

Construct	Item		Factor Loadings	VIF	AVE	CR	α
First-order							
Values of Openness to Change	VOC1	I seek constant surprises	0.904 ***	3.257	0.895	0.897	0.826
	VOC2	I have an adventurous spirit, eager to explore innovation	0.915 ***	3.39			
	VOC3	I am receptive to new experiences	0.909 ***	4.271			
Attitudes	ATT1	I believe utilizing digital technology for physical activity is a beneficial approach	0.947 ***	4.437	0.932	0.932	0.88
	ATT2	I think digital technology provides significant advantages for sports participation	0.934 ***	3.608			
	ATT3	I am convinced that digital technology enhances the value of sports participation	0.933 ***	3.722			
Adoption Intentions	AI1	I intend to incorporate digital technology into my sports participation	0.921 ***	3.257	0.924	0.926	0.868
	AI2	I anticipate utilizing digital technology in future sports participation	0.93 ***	3.39			
	AI3	I plan to embrace digital technology as a means to engage in sports activities	0.944 ***	4.271			
Self-Efficacy	SE1	If I put in my best effort, I can consistently resolve challenges in my life	0.909 ***	3.273	0.887	0.908	0.814
	SE2	Confident in handling life’s surprises effectively	0.895 ***	2.053			
	SE3	Even if others object to me, I can still achieve my goal	0.903 ***	3.156			
Usage Barrier (RAA)	UB1	Engaging in sports participation through digital technology is challenging	0.916 ***	2.987	0.908	0.909	0.845
	UB2	Digital technology may not offer sufficient convenience for sports participation	0.915 ***	2.837			
	UB3	The requirement for specialized facilities may restrict the use of digital technology in sports engagement	0.926 ***	3.231			
Risk Barrier (RAA)	RB1	Concerns about reliability arise when utilizing digital technology for sports engagement	0.902 ***	2.668	0.899	0.9	0.832
	RB2	I have concerns about the potential for information leaks when using digital technology in sports activities	0.929 ***	3.225			
	RB3	I perceive using digital technology for sports participation as a risky endeavor	0.904 ***	2.649			
Value Barrier (RAA)	VB1	I perceive little advantage in utilizing digital technology for engaging in sports activities	0.905 ***	2.651	0.895	0.895	0.827
	VB2	I believe that digital technology does not improve my sports abilities	0.91 ***	2.69			
	VB3	I see no distinct benefits in integrating digital technology into sports participation	0.912 ***	2.763			
Tradition Barrier (RAA)	TB1	For me, traditional methods of sports participation are entirely sufficient	0.913 ***	2.772	0.893	0.893	0.823
	TB2	I feel at ease engaging in sports that are familiar and have a long history	0.907 ***	2.619			
	TB3	I prefer traditional sports for greater satisfaction	0.901 ***	2.574			
Image Barrier (RAA)	IB1	I’m skeptical about using digital tech in physical activities	0.914 ***	2.933	0.917	0.918	0.858
	IB2	I find it challenging to merge sports with digital tech	0.93 ***	3.382			
	IB3	I view digital technology as intricate and complicated	0.934 ***	3.638			
Perceived Ease of Use (RFA)	PEOU1	I believe digital technology in sports can be straightforward	0.91 ***	2.866	0.9	0.902	0.834
	PEOU2	I find it straightforward to understand and apply digital technology for engaging in sports activities	0.916 ***	2.786			
	PEOU3	Digital technology enables flexible and interactive engagement in sports	0.912 ***	2.769			
Perceived Usefulness (RFA)	PU1	Digital tech speeds up sports task completion	0.929 ***	3.376	0.912	0.913	0.851
	PU2	Incorporating digital tech in sports boosts my performance and output	0.91 ***	2.8			
	PU3	Digital technology can make participating in sports more convenient and accessible	0.928 ***	3.293			
Perceived Enjoyment (RFA)	PE1	I believe digital tech simplifies sports and enhances enjoyment	0.92 ***	3.096	0.925	0.926	0.87
	PE2	Digital participation in sports is naturally enjoyable	0.94 ***	3.978			
	PE3	I am a staunch proponent of digital tech in sports participation	0.938 ***	3.879			
Second-order							
RFA	PE		0.807 ***	1.805	0.854	0.857	0.632
	PEOU		0.792 ***	1.367			
	PU		0.784 ***	1.394			
RAA	RB		0.751 ***	1.387	0.708	0.711	0.612
	TB		0.794 ***	1.697			
	UB		0.838 ***	1.864			
	VB		0.825 ***	2.125			
	IB		0.763 ***	2.017			

Note: RAA = Reasons against adoption; RFA = Reason for adoption; *** *p* < 0.001.

**Table 3:** Construct correlations and measurement properties.

	AI	ATT	VOC	SE	PE	PEOU	PU	RB	TB	UB	VB	IB
AI	***0.932***	0.545	0.492	0.226	0.402	0.184	0.12	0.207	0.227	0.491	0.303	0.334
ATT	0.507	***0.938***	0.419	0.104	0.455	0.213	0.145	0.319	0.335	0.562	0.305	0.367
VOC	0.449	0.383	***0.909***	0.031	0.26	0.092	0.096	−0.153	−0.14	−0.257	−0.118	−0.142
SE	0.205	0.099	0.033	***0.902***	0.025	0.059	0.033	−0.07	0.084	0.205	0.167	−0.109
PE	0.372	0.423	0.285	0.04	***0.933***	0.486	0.479	0.16	0.197	0.336	0.138	−0.101
PEOU	0.166	0.196	0.102	0.067	0.445	***0.913***	0.503	0.054	0.092	0.203	0.125	−0.07
PU	0.111	0.134	0.106	0.041	0.44	0.457	***0.922***	0.097	0.117	0.179	0.091	−0.075
RB	−0.188	−0.292	0.169	0.076	−0.146	−0.047	−0.088	***0.912***	0.623	0.579	0.603	0.393
TB	−0.207	−0.306	0.157	−0.071	−0.179	−0.083	−0.106	0.559	***0.907***	0.619	0.655	0.461
UB	−0.451	−0.517	0.284	−0.189	−0.308	−0.184	−0.162	0.524	0.558	***0.919***	0.649	0.622
VB	−0.275	−0.279	0.131	−0.155	−0.126	−0.112	−0.082	0.542	0.586	0.585	***0.909***	0.556
IB	−0.308	−0.34	0.157	0.119	0.109	0.081	0.082	0.433	0.509	0.681	0.614	***0.926***

Note: The diagonal shows the square root of the AVE in italics/bold; values above the diagonal are the HTMT values.

**Table 4:** Hypothesis results.

**First-Order Paths**										
**Paths**				**β**	**T**	**Result**	**VIF**	**f^2^**	**LLCI**	**ULCI**
H1a	VOC	→	RFA	0.223	4.032 ***	Yes	1.009	0.059	0.114	0.332
H1b	VOC	→	RAA	−0.217	3.967 ***	Yes	1.009	0.052	−0.322	−0.109
H2	VOC	→	ATT	0.287	5.465 ***	Yes	1.328	0.1	0.185	0.391
H3a	RFA	→	ATT	0.148	2.677 **	Yes	1.292	0.027	0.041	0.256
H3b	RAA	→	ATT	−0.296	6.033 ***	Yes	1.153	0.122	−0.392	−0.2
H4a	RFA	→	AI	0.14	3.198 **	Yes	1.364	0.024	0.054	0.226
H4b	RAA	→	AI	−0.136	2.971 **	Yes	1.296	0.024	−0.23	−0.05
H5	ATT	→	AI	0.23	4.357 ***	Yes	1.848	0.048	0.125	0.33
H6	VOC	→	AI	0.296	6.265 ***	Yes	1.639	0.089	0.205	0.389
H7a	SE x VOC	→	RFA	0.342	7.185 ***	Yes	1.008	0.151	0.248	0.432
H7b	SE x VOC	→	RAA	−0.166	3.346 **	Yes	1.008	0.033	−0.265	−0.068
H7c	SE x VOC	→	ATT	0.222	4.502 ***	Yes	1.204	0.071	0.124	0.318
H7d	SE x RFA	→	ATT	0.095	1.879	NO	1.206	0.014	−0.001	0.198
H7e	SE x RAA	→	ATT	0.049	0.944	NO	1.092	0.003	−0.052	0.151
H7f	SE x RFA	→	AI	0.116	2.379 *	Yes	1.275	0.02	0.02	0.213
H7g	SE x RAA	→	AI	−0.019	0.368	NO	1.211	0.001	−0.123	0.083
H7h	SE x ATT	→	AI	−0.115	2.105 *	Yes	1.569	0.014	−0.227	−0.01
H7i	SE x VOC	→	AI	0.038	0.694	NO	1.428	0.002	−0.064	0.148
**Second-Order Paths**										
**Paths**				**β**	**T**	**LLCI**	**ULCI**			
RFA		→	PEOU	0.792	35.004 ***	0.744	0.832			
RFA		→	PU	0.784	28.927 ***	0.726	0.832			
RFA		→	PE	0.807	34.962 ***	0.756	0.847			
RAA		→	UB	0.838	47.371 ***	0.801	0.871			
RAA		→	RB	0.751	24.502 ***	0.688	0.806			
RAA		→	VB	0.825	42.95 ***	0.784	0.861			
RAA		→	TB	0.794	35.794 ***	0.748	0.834			
RAA		→	IB	0.763	28.443 ***	0.707	0.812			

* *p* < 0.05; ** *p* < 0.010; *** *p* < 0.001.

**Table 5:** Results of R^2^ and predictive relevance Q^2^.

	R^2^	Q^2^
ATT	0.379	0.324
AI	0.4	0.335
RAA	0.094	0.056
RFA	0.165	0.098
VOC	-	-
SE	-	-

## Data Availability

The raw data supporting the conclusions of this article will be made available by the authors on request.
